# A New Synthetic Allotetraploid (A_1_A_1_G_2_G_2_) between *Gossypium herbaceum* and *G*. *australe*: Bridging for Simultaneously Transferring Favorable Genes from These Two Diploid Species into Upland Cotton

**DOI:** 10.1371/journal.pone.0123209

**Published:** 2015-04-16

**Authors:** Quan Liu, Yu Chen, Yu Chen, Yingying Wang, Jinjin Chen, Tianzhen Zhang, Baoliang Zhou

**Affiliations:** State Key Laboratory of Crop Genetics & Germplasm Enhancement, MOE Hybrid Cotton R&D Engineering Research Center, Nanjing Agricultural University, Nanjing, Jiangsu, People’s Republic of China; CIRAD, FRANCE

## Abstract

*Gossypium herbaceum*, a cultivated diploid cotton species (2n = 2x = 26, A_1_A_1_), has favorable traits such as excellent drought tolerance and resistance to sucking insects and leaf curl virus. *G*. *australe*, a wild diploid cotton species (2n = 2x = 26, G_2_G_2_), possesses numerous economically valuable characteristics such as delayed pigment gland morphogenesis (which is conducive to the production of seeds with very low levels of gossypol as a potential food source for humans and animals) and resistance to insects, wilt diseases and abiotic stress. Creating synthetic allotetraploid cotton from these two species would lay the foundation for simultaneously transferring favorable genes into cultivated tetraploid cotton. Here, we crossed *G*. *herbaceum* (as the maternal parent) with *G*. *australe* to produce an F_1_ interspecific hybrid and doubled its chromosome complement with colchicine, successfully generating a synthetic tetraploid. The obtained tetraploid was confirmed by morphology, cytology and molecular markers and then self-pollinated. The S_1_ seedlings derived from this tetraploid gradually became flavescent after emergence of the fifth true leaf, but they were rescued by grafting and produced S_2_ seeds. The rescued S_1_ plants were partially fertile due to the existence of univalents at Metaphase I of meiosis, leading to the formation of unbalanced, nonviable gametes lacking complete sets of chromosomes. The S_2_ plants grew well and no flavescence was observed, implying that interspecific incompatibility, to some extent, had been alleviated in the S_2_ generation. The synthetic allotetraploid will be quite useful for polyploidy evolutionary studies and as a bridge for transferring favorable genes from these two diploid species into Upland cotton through hybridization.

## Introduction

The genus *Gossypium* comprises 50 species, including four cultivated cotton species, i.e., two diploids (*G*. *arboreum*, *G*. *herbaceum*, 2n = 26) and two tetraploids (*G*. *hirsutum*, *G*. *barbadense*), as well as 46 wild species [1]. Approximately 90% of commercially produced cotton comes from Upland cotton, the species *G*. *hirsutum* L. However, numerous studies have shown that Upland cotton has a low level of genetic diversity, which it has been losing over the past century [2–8] due to the overuse of relatively few cultivars in larger areas for both breeding and production [9]. Thus, the existing genetic base of cotton should be broadened to adapt to various adverse conditions including biotic and abiotic stress. Although these wild species are short-fibered or lintless, they have a number of desirable traits, such as fiber quality and resistance to salt, heat, drought, insects and diseases [10–12].

*G*. *australe* F. Mueller, a wild diploid cotton species (2n = 2x = 26, G_2_G_2_) (with short brownish straightly spreading fibers) that is native to Australia, possesses numerous economically valuable characteristics such as delayed pigment gland morphogenesis, which is conducive to the production of seeds with very low levels of gossypol as a potential source of food and feed for human and animal consumption, resistance to pest insects (aphids and mites) and diseases (*Fusarium* and *Verticillium* wilt) and tolerance to abiotic stress (drought); these traits would be useful if transferred into the most important tetraploid cultivated species, *G*. *hirsutum* L. (2n = 4x = 52, AADD). *G*. *herbaceum* L., a cultivated diploid cotton species (2n = 2x = 26, A_1_A_1_) that is native to Western China and adjacent regions of the former Soviet Union, have favorable traits, such as high tolerance to drought and resistance to sucking pests (hoppers, white flies, thrips, aphids) and leaf curl virus [13,14]. There are three strategies that could be used to simultaneously introduce desirable genes from these two species into cultivated tetraploid cotton.

The first strategy (pentaploid pathway) is to make two crosses at the beginning, i.e., one cross between diploid species A and a tetraploid cotton line, the other between diploid species B and the same tetraploid cotton line for the production of two triploid interspecific hybrid F_1_s. Then the chromosome complement of the two triploids will be doubled to generate two hexaploids. The two hexaploids can be backcrossed with the tetraploid parent to produce two pentaploids, and then backcrossed repeatedly to the tetraploid parent to develop introgression lines, respectively [15]. Finally, inter-mating between introgression lines should be made to pyramid desirable genes both from species A and B. So, the pentaploid pathway is laborious and time-consuming. The second strategy (hexaploid pathway) is to cross one diploid species with a tetraploid cotton line, double the chromosome complement, and again cross another diploid species toward the tetraploid level and backcross repeatedly to the tetraploid parent [16–18]. The hexaploid pathway is labor-saving but time-consuming. The third strategy (tetraploid pathway) involves hybridizing two different genomic diploid species. Once the chromosome complement of this diploid hybrid is doubled, the hybrid is crossed with a cultivated tetraploid line to generate a trispecies hybrid, which is then repeatedly backcrossed with the cultivated tetraploid [19–21]. Using the third strategy, the triple hybrid strains (*G*. *arboreum* L. × *G*. *thurberi* Todaro × *G*. *hirsutum* L.) became the foundation of Pee Dee germplasm, which are well known to represent an array of genetic diversity in Upland cotton in the United States [22,23]. The use of the tetraploid pathway may simplify this procedure and shorten the time required to simultaneously introduce desirable genes from two species. To our knowledge, as yet, there is no report that allotetraploid (A_1_A_1_G_2_G_2_) between *Gossypium herbaceum* and *G*. *australe* have been successfully obtained.

In this study, because it is quite difficult to directly cross *G*. *herbaceum* and *G*. *australe* with the tetraploid *G*. *hirsutum* due to interspecific incompatibility, we employed the tetraploid pathway to produce an interspecific hybrid to bridge for simultaneously transferring desirable genes from these two diploid species into Upland cotton. Here, *G*. *herbaceum* is used as maternal parent to cross with *G*. *australe* to produce interspecific F_1_ hybrid (2n = A_1_G_2_ = 26). To overcome its sterility, the branches from the verified F_1_ interspecific hybrid were treated with colchicine solution to double its chromosome complement to generate tetraploidy, which can be further confirmed by morphological and molecular cytological observation.

## Materials and Methods

### Plant materials

The Asiatic diploid cultivated cotton *Gossypium herbaceum* race *kuljianum* cv Hongxingcaomian (2n = 2x = 26, A_1_A_1_), a highly inbred line, and an Australian diploid wild species, *G*. *australe* F. Mueller (2n = 2x = 26, G_2_G_2_), were obtained from Hainan Wild Cotton Growing Garden, Cotton Research Institute of Chinese Academy of Agricultural Sciences.

### Methods

#### Interspecific hybridization

Flowers of *G*. *herbaceum* were emasculated by hand in the afternoon before anthesis, and each stigma was covered with a 5 cm length of plastic straw to prevent cross-hybridization. The following morning, stigmas were pollinated by hand using fresh pollen from *G*. *australe* between 9 am and noon under natural field conditions in Hainan province, China; the pollinated flowers were individually tagged. The obtained putative interspecific hybrid seeds were sown in nursery pots and the seedlings were transplanted into ceramic pots at Jiangpu Breeding Station, Nanjing Agricultural University (JBS/NAU), in 2007. In the winter, all plants were moved into a greenhouse at Pailou Experimental Station (PES), NAU.

#### Colchicine treatment

In 2007–2012, the interspecific hybrid plants were preserved and propagated by grafting. The stem apices of vegetative branches from these hybrid plants were immersed in 0.10% (w/v) colchicine solution for 24 h and grown in ceramic pots under natural conditions.

#### Chromosome preparation for cytological observations

Root tips were used for mitotic metaphase chromosome preparation, and young buds approximately 3–4 mm long were used for meiotic metaphase chromosome preparation. Mitotic chromosome preparation was performed as described by Wang et al [24] with some modifications. Root tips were cut from S_1_ and S_2_ germinated seeds that had been treated with 25 μg/ml cycloheximide at 29°C for 2 h to accumulate metaphase cells. The root tips were then fixed in a solution of ethanol: acetic acid (3:1 v/v; Carnoy's Fluid) for 24 h. After fixation, the root tips were macerated in 4% cellulase and 1% pectinase at 37°C for 40 min and fixed in Carnoy's Fluid for more than 2 h; the treated root tips were stored in 70% ethanol at -20°C. The treated root tips were squashed onto slides in a drop of 45% acetic acid to make the tip cells disperse and to allow the metaphase-chromosomes to spread out. Slides with easily visualized cells in metaphase were stored at -70°C for more than 12 h until use for GISH analysis. Prior to analysis, after the slides were transferred from -70°C storage, and the cover slips were immediately removed, and the samples were dehydrated in 100% ethanol for 5 min.

Meiotic metaphase chromosomes from S_1_ plants were prepared as previously described by Chen et al [25]. Young flower buds were collected between 8 and 10 am and fixed in ethanol-chloroform-acetic acid (6:3:1) fixative for 2–24 h at 4°C. Next, the buds were screened for cells in Metaphase I, and several anthers from the selected bud were placed onto an ethanol-washed glass slide with a drop of 45% acetic acid (v/v), freed of debris and squashed. The material was observed through a 100× objective lens under an Olympus BX51 microscope.

#### Genomic in situ hybridization (GISH)

Genomic DNA was extracted from *G*. *australe* as described by Paterson et al [26] and labeled with digoxigenin-11-dUTP (Roche Diagnostics, Mannheim, Germany) by nick translation. The labeled DNA probe fragments were between 200 and 500 bp long. Fluorescence *in situ* hybridization was carried out as described by Wang et al [24] with some modifications. Somatic mitotic S_2_ (progenies from S_1_ self-pollinated) cells were used as targets. Chromosomal DNA was denatured by placing the slides in 50 mL 70% formamide, 2x SSC at 72°C for 2.5 min and immediately dehydrating them in an ethanol series at −20°C, followed by air-drying. Fifteen microliters of a mixture containing 25–50 ng labeled DNA, 50% (w/v) dextran sulfate, 10 μg sheared salmon sperm DNA, an appropriate amount of sheared cotton DNA as blocking DNA (probe: blocking DNA = 1:100) and 1.5 μL 20× SSC was denatured at 97°C for 10 min, chilled on ice, annealed at 37°C for 1 h and applied to a dry slide. Following overnight incubation at 37°C, the coverslips were removed and the slides were washed at increasing stringency by rinsing twice at 43°C in 2x SSC for 5 min, once in 2x SSC, 60% formamide for 13 min, twice more in 2x SSC for 5 min and once in 1x PBS for 5 min. Probes were detected with 20 μg/mL rhodamine-conjugated anti-digoxigenin antibody (Roche Diagnostics). The slides were stained in 4’,6-diamidino-2-phenylindole (DAPI; Diagnostics) for 10 min at room temperature, and anti-fade (Vector, USA) was applied under the coverslip. The slides were examined, and more than 20 images of well-spread somatic chromosomes at metaphase were obtained for each individual using an Olympus BX51 fluorescence microscope. Chromosome and FISH signal images were captured using an Evolution VF CCD camera (Media Cybernetics, Bethesda, MD, USA) and merged using Image-Pro Express software (Media Cybernetics, Bethesda, MD, USA).

#### SSR molecular marker identification

A total of 658 simple sequence repeat (SSR) primer pairs were randomly chosen according to our cotton genetic maps [27] and used to screen the parents for polymorphisms. The obtained polymorphic primers were employed to verify the authenticity of the putative interspecific F_1_ hybrid and its derivatives (the new synthetic tetraploid, S_1_). These SSR primer sequences are available at http://www.cottonmarker.org. SSR-PCR amplifications were performed using a Programmable Thermal Controller (MJ Research), and PCR product electrophoresis and silver staining were conducted as described by Zhang et al [28,29].

#### Morphological observations

The shapes and sizes of fully expanded leaves from the same position on the parents, the hybrid plants and its derivatives (the new synthetic tetraploid, S_1_) were characterized. Floral morphological traits were observed on the day of anthesis.

## Results

### Production of a new synthetic allotetraploid between *Gossypium herbaceum* and *Gossypium australe*

A total of 16 putative hybrid seeds were obtained from 200 pollinated flowers and planted in nursery pots and the seedlings were transplanted into ceramic pots at JBS/NAU in 2007. At the maturity stage, only one plant resembled *G*. *australe*, which was a putative F_1_ interspecific hybrid (2n = 2x = A_1_G_2_ = 26), while the morphological traits of the other plants were similar to those of the maternal parent; these lines were *G*. *herbaceum* plants, not F_1_ hybrids. The putative interspecific F_1_ hybrid plant appeared to be highly male and female sterile, as no pollen was released and no bolls were produced when the plant was pollinated by *G*. *herbaceum*. The sole putative hybrid plant was propagated by grafting to >20 plants for prevention of the interspecific hybrid from lost due to the death caused by colchicine treatment, and the stem apices of these grafted plants were treated with 0.10% colchicine for 24 h during squaring stage. During the first five years, no boll was produced and no seed was obtained for these grafted hybrid plants. Finally, in the sixth year, one branch of the hybrid plant had produced three bolls (S1 Fig), and a total of 19 S_1_ seeds were obtained from these bolls (S2 Fig) by cleistogamy or self-pollination in 2012. The results demonstrate that the chromosome complement of the branch from this hybrid plant could be doubled to the tetraploid level (2n = 4x = A_1_A_1_G_2_G_2_ = 52) and the interspecific tetraploid hybrid had partial fertility. In the winter, all of the grafted F_1_ hybrid plants were preserved in a greenhouse at PES/NAU.

In 2013, we planted three S_1_ seeds (derived from the grafted hybrid F_1_) on MS medium [30] in a triangular flask to collect healthy root tips (normally expanded and elongated) for cytological observation. However, the seeds germinated very slowly and no healthy root tips were collected. Thus, we planted the S_1_ seedlings on soil in small plastic pots. Initially, the first four true leaves of the S_1_ seedlings were dark green (S3 Fig); however, the seedlings gradually became flavescent beginning at the top leaves, followed by the lower leaves after the fifth true leaf emerged (S4 Fig). We selected one stem apex from one S_1_ seedling to graft onto a *G*. *barbadense* plant to rescue the interspecific hybrid. Finally, the grafted S_1_ plant was able to further grow and set S_2_ seeds, while the other two S_1_ seedlings died two weeks later, probably due the cessation of root growth resulting from interspecific incompatibility. The rescued S_1_ plants propagated by vegetative fashion (grafting), however, were partially fertile, as only 22 seeds were obtained from a total of five bolls from the three grafted S_1_ plants (derived from vegetative production of the S_1_ by grafting).

**Fig 1:**
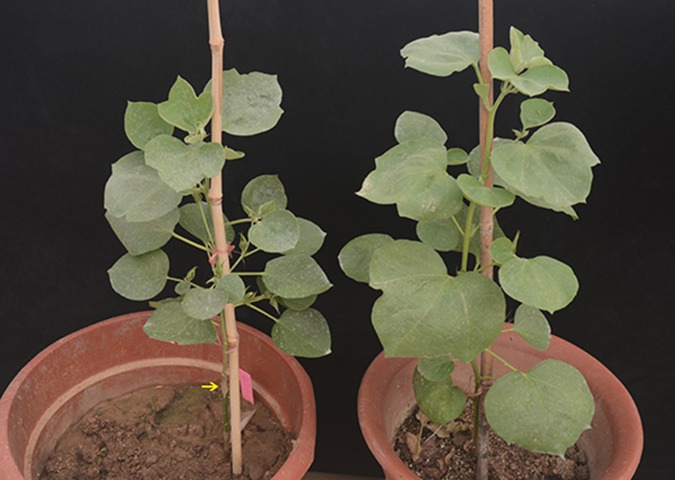
Grafted S_1_ plant (left) and non-grafted S_2_ seedling (right) grew normally. The arrow indicates the grafted part of the plant shown on the left.

In 2014, we planted three S_2_ seeds on MS medium in a triangular flask to collect healthy root tips for cytological observation. The seeds germinated normally, and root tips were collected. These S_2_ plants grew well into the squaring stage (Fig 1). No flavescent lethal phenomenon occurred during the development of S_2_ plants, which implies that interspecific incompatibility, to some extent, had been alleviated in the S_2_ generation. The above results suggest that both S_1_ and S_2_ were new synthetic allotetraploid plants.

### Validation of a new synthetic allotetraploid between *Gossypium herbaceum* and *G*. *australe* by chromosome observation

We further confirmed the identity of the synthetic allotetraploid using four techniques, namely, molecular cytogenetic discrimination by GISH, observation of chromosome association at meiosis, morphological observation and molecular marker identification.

#### Genome component analysis of the synthetic allotetraploid by genomic in situ hybridization (GISH)

**Fig 2:**
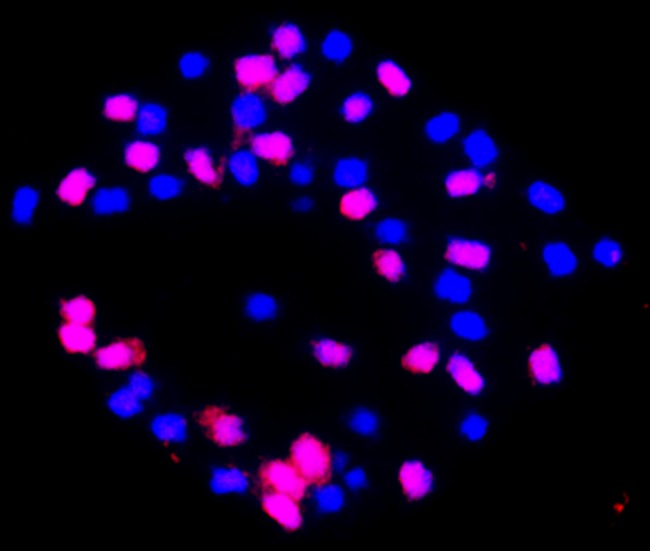
Genomic *in situ* hybridization showing the chromosome components of an S_2_ mitotic cell of the new synthetic allotetraploid (Slides were stained in 4’, 6-diamidino-2-phenylindole [DAPI]). Red signals indicate the 26 chromosomes of *G*. *australe* (probe labeled with digoxigenin-11-dUTP) and blue signals indicate the 26 chromosomes of *G*. *herbaceum* (stained with DAPI).

To confirm the authenticity of the synthetic allotetraploid and to examine its genome components, we performed GISH under standard stringency conditions using G_2_ gDNA from *G*. *australe* labeled with digoxigenin (DIG)-Nick Translation Mix DNA as a labeled probe and A_1_ gDNA from *G*. *herbaceum* race *kuljianum* cv Hongxingcaomian as unlabeled DNA. Somatic S_2_ mitotic cells were used as targets. Red hybridization signals were consistently detected on 26 chromosomes (G_2_ genome) and blue signals were detected on the other 26 chromosomes (A_1_ genome) in over 30 well-spread somatic chromosome cells that were observed. Thus, the chromosomes from the two genomes could clearly be differentiated based on color (Fig 2). Therefore, the authenticity of the new synthetic allotetraploid has been confirmed and its genome components can be readily discriminated as well.

#### Chromosome associations at meiosis of PMCs from the synthetic allotetraploid

**Fig 3:**
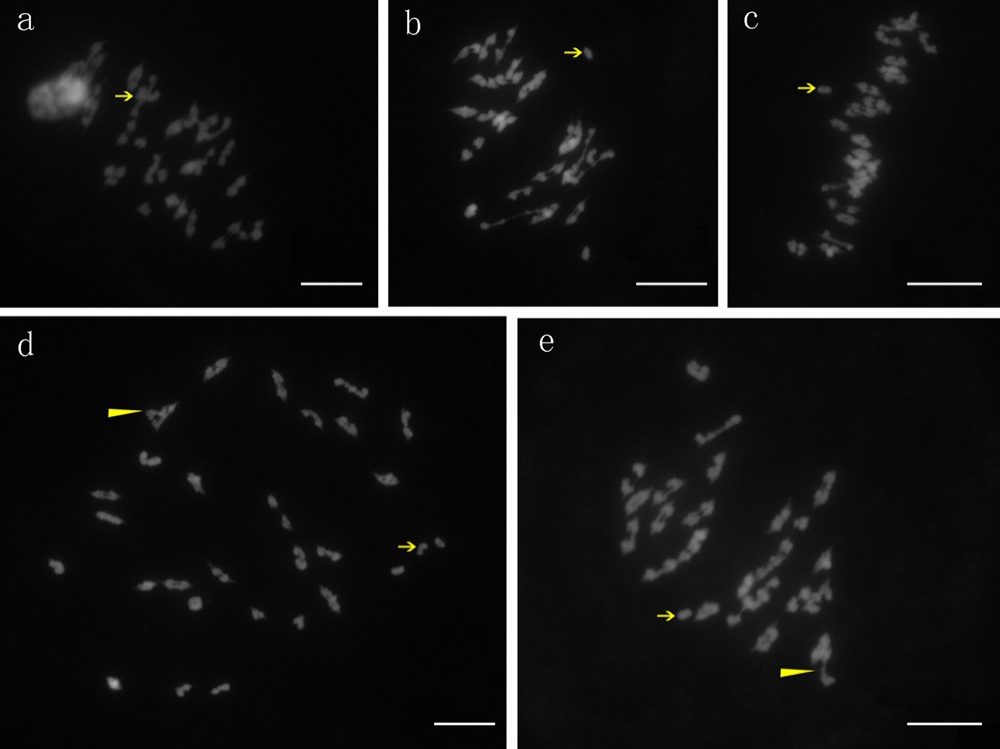
Chromosome associations in PMCs at Metaphase I of meiosis. a, 25II + 2I; b, 24II + 4I; c, 25II + 2I; d, 23II + 3I + III; e, 24II + I + III. Arrows indicate the univalents and arrowheads indicate the trivalents. Bar = 10 μm.

We then performed cytological observations of the new synthetic allotetraploid of *G*. *herbaceum* × *G*. *australe*. All of the cells observed had 52 chromosomes, indicating that they were tetraploid (2n = 4x = 52; Table 1, Fig 3), which further confirmed the authenticity of the new synthetic allotetraploid. The chromosome configurations in the synthetic allotetraploid were variable, with uni-, bi- and trivalents. Of the 49 pollen mother cells observed, most cells (24/49) had two univalents and 25 bivalents, followed by cells (14/49) with four univalents and 24 bivalents. Some cells (9/49) contained three univalents, 23 bivalents and one trivalent. Only two cells had 26 bivalents. The average chromosome configurations were 2.67 uni-, 23.33 bi- and 0.18 trivalents. The number of univalents ranged from zero to four, with two being the most frequent number, followed by four and three. The number of bivalents ranged from 23 to 26, with 25 being the most frequent number followed by 23 and 24 (Fig 3). The high frequency of univalents in pollen mother cells (PMC) at Metaphase I in meiosis explained why the synthetic allotetraploid plants were partially fertile, since due to disordered segregation, univalents were often lost at Anaphase I, leading to the formation of unbalanced, nonviable gametes lacking a complete set of chromosomes.

**Table 1:** Chromosome configurations of pollen mother cells at Metaphase I of meiosis.

No. of PMCs	I	II	III	No. of chromosomes
24	2	25		52
14	4	24		52
9	3	23	1	52
2		26		52
Range	0~4	23~26	0~1	52
Average	2.67	23.33	0.18	52

#### Morphological traits of the synthetic allotetraploid

**Fig 4:**
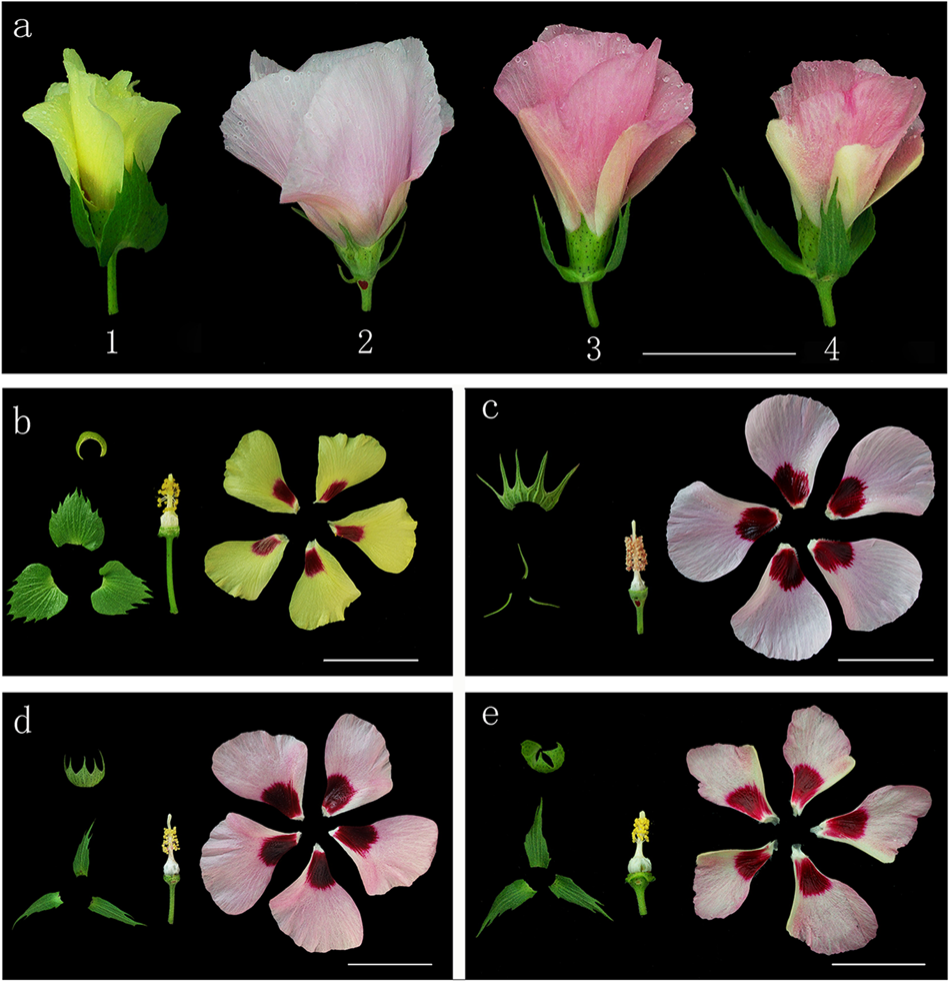
Flowers of the new synthetic allotetraploid and its parents. a-1, *G*. *herbaceum*; a-2, *G*. *australe*; a-3, interspecific hybrid F_1_; a-4, S_1_. b, *G*. *herbaceum*; c, *G*. *australe*; d, interspecific hybrid F_1_; e, S_1._ Bar = 25 mm.

**Fig 5:**
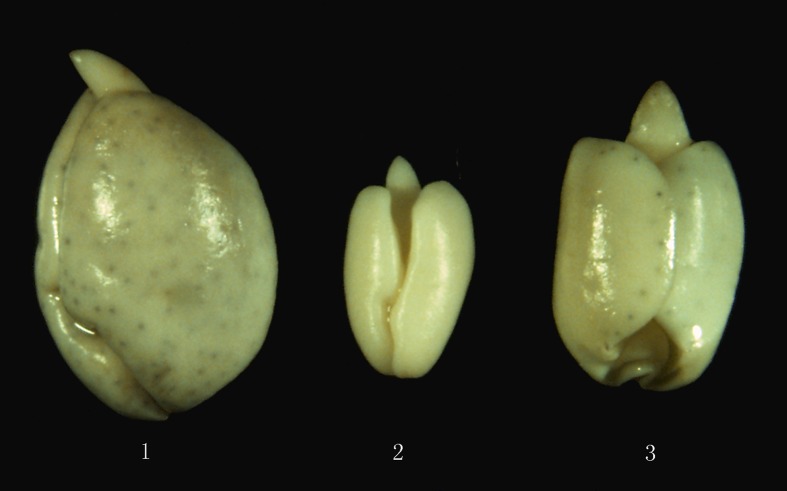
Seed kernels of *G*. *herbaceum* (1), *G*. *australe* (2) and the new synthetic allotetraploid S_1_ (3). Numerous pigment glands on the cotyledon surface of *G*. *herbaceum* (1); no pigment glands on the cotyledon surface of *G*. *australe* (2); a few pigment glands on the cotyledon surface margin of the synthetic allotetraploid S_1_ (3).

**Fig 6:**
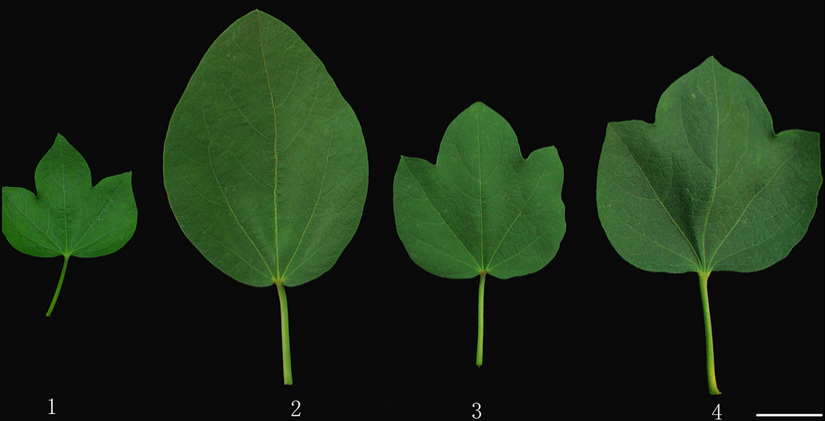
Leaf shapes from left to right: *G*. *herbaceum* (1), *G*. *australe* (2) and the synthetic allotetraploid S_1_ (3) and S_2_ (4). Bar = 25 mm.

We examined 24 morphological traits of the synthetic allotetraploid (S_1_) and its parents during flowering, including the color, shape and size of leaves, flowers and so on (Table 2). The results indicate that most traits of the interspecific hybrid F_1_ plant tended to resemble those of the paternal parent, *G*. *australe*, e.g., hairiness of leaves and stems, petal color and fibers. A few traits resembled those of the maternal parent, *G*. *herbaceum*, e.g., anther color. Some traits exhibited intermediate phenotypes, e.g., bract shape, calyx size and the number of calyx teeth, leaf lobes, anthers, seed pigment glands and fiber (Figs 4 and 5, S2 Fig). Many characters of S_1_ plants, the new synthetic allotetraploid, were similar to those of the F_1_, while S_1_ plants had larger dark green leaves, which was the most distinctive trait from that of the F_1_ (Fig 6).

**Table 2:** Morphological characters of interspecific hybrid F_1_, S_1_ and their parents.

Characters	*G*. *herbaceum*	*G*. *australe*	F_1_	S_1_
**Stem color**	Green	Grey green	Green	Green
**Stem hairiness**	Pilose	Pubescence	Pubescence	Pubescence
**Leaf color**	Green	Grey green	Green	Dark green
**Leaf hairiness**	Pilose	Pubescence	Pubescence	Pubescence
**Leaf shape**	Deeply lobed	Very few lobed	Deeply lobed	Deeply lobed
**Leaf lobation**	2–4 broad shallow	0–1	0–4 narrow deep	0–4 narrow deep
**Leaf texture**	Thin soft	Thick soft	Thin soft	Thick hard
**Petiol color**	Green	Grey green	Green	Deep green
**Leaf length(cm)**	5.55±0.40	7.54±0.35	6.43±0.43	6.56±0.43
**Leaf width(cm)**	6.71±0.32	6.00±0.49	6.23±0.57	6.38±0.43
**Petiol length (cm)**	3.75±0.44	3.1±0.23	3.41±0.42	3.48±0.52
**Petal color**	Yellow	Lilac	Pink	Dark pink
**Flower size**	Small	Small	Small	Small
**Petal spot**	Dark red	Dark red	Dark red	Dark red
**Petal length (cm)**	2.35±0.21	3.72±0.16	3.59±0.22	3.61±0.23
**Petal width (cm)**	2.19±0.24	3.46±0.19	3.35±0.38	3.36±0.32
**Stigma color**	Creamy	Creamy	Creamy	Creamy
**Stigma length (cm)**	0.38±0.08	0.62±0.10	0.49±0.10	0.48±0.12
**Anther color**	Yellow	White	Light yellow	yellow
**Anther number**	48.30±7.32	107.00±2.14	67.70±8.42	54.50±6.50
**Bracteole dentation**	6–12 deep	3 acicular	2–4 deep	2–4 deep
**Bracteole length (cm)**	1.70±0.10	1.14±0.10	1.90±0.16	1.68±0.13
**Bracteole width (cm)**	1.40±0.16	Very narrow	0.61±0.08	0.49±0.07
**Pedicel length (cm)**	0.88±0.12	0.95±0.10	1.01±0.19	1.00±0.16

#### Examination of the synthetic allotetraploid using SSR molecular markers

**Fig 7:**
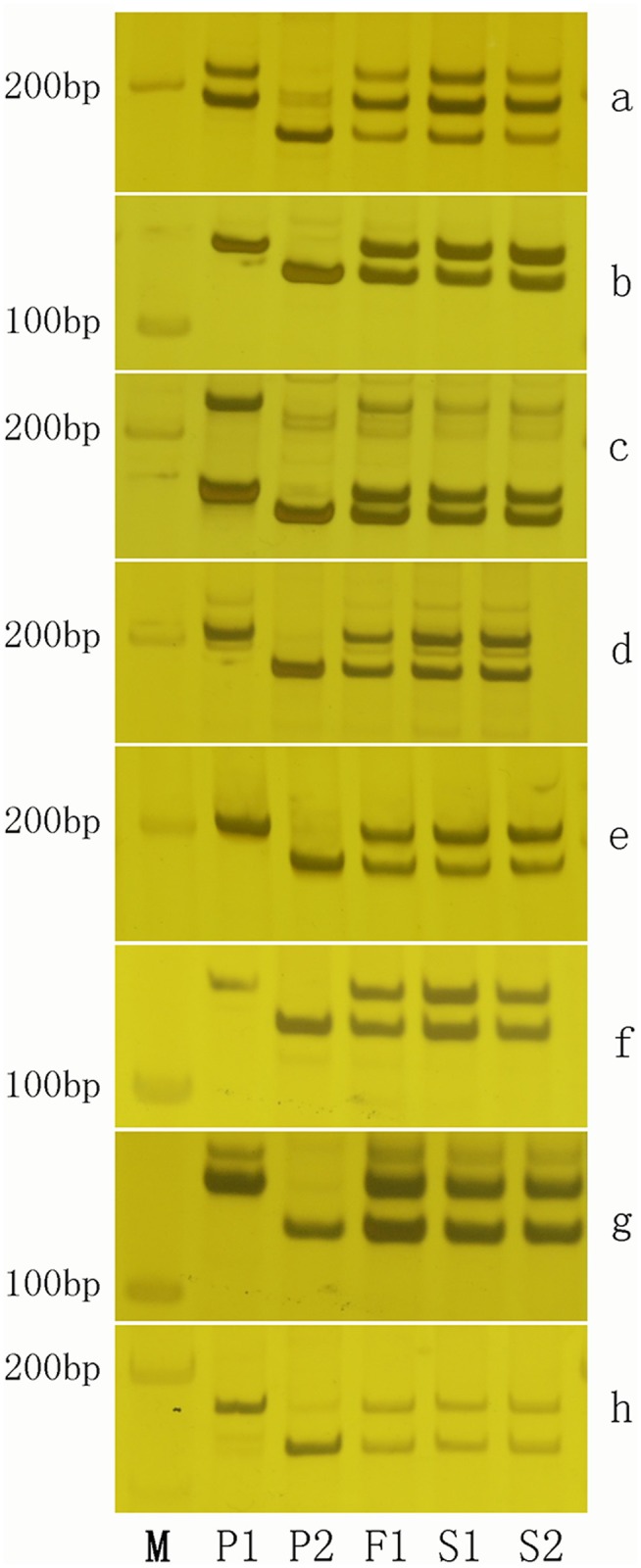
Validation of the new synthetic allotetraploid of *G*. *herbaceum* × *G*. *australe* using a randomly selected set of polymorphic SSR primers. From a to h, polymorphic amplicons of F_1_, S_1_, S_2_ and its parents were detected using SSR primers NAU207, NAU704, NAU905, NAU2325, NAU3995, NAU7751, NAU7699 and NAU6064, respectively. Here, showed eight codominant markers. P1, *G*. *herbaceum*; P2, *G*. *australe*; F_1_, *G*. *herbaceum* × *G*. *australe*; S_1_, synthetic allotetraploid of *G*. *herbaceum* × *G*. *australe*; S_2_, progeny from S_1_ self-pollination; M, molecular marker sizes (100 bp ladder).

We used a total of 658 SSR primer pairs/combinations that were randomly selected at genetic intervals of 5–10 cM (genome coverage of ~90%) from the linkage map of the *G*. *hirsutum* and *G*. *barbadense* genome constructed at our institute [27] to screen polymorphic primers between *G*. *herbaceum* and *G*. *australe* and to confirm the authenticity of the synthetic allotetraploid. Approximately 70% (459/658) of the SSRs detected polymorphisms between these two species, showing a very high diversity at SSR molecular marker level. Of the 459 pairs of polymorphic primers used to characterize the new synthetic allotetraploid, 212 (46%) showed codominance in the synthetic allotetraploid, whereas 140 (31%) were dominant in *G*. *herbaceum* and 107 (23%) were dominant to *G*. *australe*. The amplicons generated using codominant/ dominant primers in the synthetic allotetraploid demonstrated that it had DNA bands from both/paternal parent(s), further confirming that the synthetic allotetraploid was derived from *G*. *herbaceum* and *G*. *australe* (Fig 7).

## Discussion

The transfer of genes of interest from wild species has played an important role in cotton breeding, and great progress has been made in the introgression of desirable traits such as superior fiber quality (length, strength and fineness) and disease resistance [22,23,31]. Wild cotton species are a valuable reservoir of agronomically useful genes and genes conferring resistance to pests and diseases [32,33]. However, wild diploid cotton species have not been fully exploited to broaden the existing narrow genetic base through distant crosses with the world’s major cultivated tetraploid cotton due to both pre- and post-fertilization barriers between these species [20,21,34,35]. In this study, not only did we successfully obtain an interspecific hybrid F_1_ and double its chromosome complement with colchicine, but we also alleviated the incompatibility of the new synthetic tetraploid (A_1_A_1_G_2_G_2_) by grafting, which will lay a solid foundation for the transfer of genes of interest from the two parental diploid species. Theoretically, the new synthetic allotetraploid has integrated favorable genes of interest, such as fiber quality, resistance to pest insect and diseases and tolerance to drought and heat, both from *G*. *herbaceum* and *G*. *australe*, which will enable us to simultaneously transfer them into Upland cotton through hybridization in the future.

Moreover, during the development of the synthetic tetraploid, we found that the chromosome complement at meiosis in PMCs did not always comprise 26 bivalents, but there was a high frequency of univalents, which is consistent with the observations on another synthetic tetraploid between *G*. *arboreum* and *G*. *bickii* [36]. Univalents are often lost at Anaphase I due to distorted segregation, leading to the formation of unbalanced, nonviable gametes lacking a complete set of chromosomes. Therefore, the fertility of synthetic tetraploids is often quite low, especially in the early generation, for example, the A_1_A_1_G_2_G_2_ tetraploid produced in this study. Based on our previous studies on diverse synthetic polyploids through cotton interspecific hybridization, however, we find that as the selfing generations of synthetic polyploids advance, the percentage of PMCs with univalents will decrease and the fertility of the artificial synthetic tetraploid will increase (data not show); the reasons for this are unclear. The mechanisms of genome evolution when two cell nuclei unite in a cotton line remain poorly understood despite the fact that numerous studies have focused on this issue [37–40]. Therefore, the synthetic cotton tetraploid produced in this study will be useful for elucidating the mechanisms of evolution in polyploids.

Furthermore, we found that the flavescent lethal phenomenon emerged in the S_1_ generation, which represents another type of interspecific incompatibility, but the plant was rescued by grafting and the S_1_ seedlings grew well and set S_2_ seeds. This flavescent lethal phenomenon might be caused by the cessation of root expansion and elongation, but it only occurred in the S_1_ (not the S_2_) generation. The mechanisms that caused the alleviation of interspecific incompatibility in S_2_ generation remain unclear. By contrast, root tip wilting was also observed by Li et al [36] in the early generation of the amphiploid *G*. *arboreum* (A genome) × *G*. *bickii* (G genome), who found that seeds germinated, but there was no further root elongation, in the absence of treatment. When the germinated seeds were treated with rooting powder solution, only a few seedlings grew. Only after several consecutive generation selections had the interspecific incompatibility been gradually alleviated [41]. The mechanisms of these two root tip wilting phenomena appear to be different. Thus, the obtained S_1_ and S_2_ seeds can be used to explore the mechanism underlying the flavescent lethal phenomena, which will increase our understanding of the basic physiology and genetics of flavescent lethal.

In addition, *G*. *australe* possesses unique valuable interesting characteristic—delayed pigment gland morphogenesis or high-gossypol cotton plants with low- gossypol seeds, which is conducive to the production of seeds with very low levels of gossypol as a potential source of food and feed for human and animal consumption. During the recent decades, however, several crosses were made between *G*. *hirsutum* and Australian species (*G*. *sturtianum*, *G*. *australe*, *G*. *bickii*) [42–52] or *G*. *arboreum* and *G*. *bickii* [53,54], the potential for use in commercial cotton production have not yet been reached. The failure to develop cotton varieties with delayed pigment gland morphogenesis has to do with poor understanding of complex genetic mechanisms, or labile gossypol content in cotton seeds. Moreover, no or very little homoeologous recombination occurrence between distant species under natural conditions also hinder the advances in cotton improvement. On the basis of genetic analysis by Zhu et al [54], the trait of high-gossypol plants with low- gossypol seeds from *G*. *bickii*, another Australian wild G genome species, is controlled by a gene located at the *Gl*_*2*_ locus, which has been temporarily named *Gl*_*2*_^*b*^. This gene, *Gl*_*2*_^*b*^, is dominant to upland cotton pigment gland alleles *Gl*_*2*_ and *gl*_*2*_ in A subgenome, but is recessive and epistatic to another pigment gland gene *Gl*_*3*_ in D subgenome. Based on above results, Chen et al [25] also explained why no *G*. *hirsutum*-*G*. *australe* alien chromosome addition line was detected to exhibit the trait of high-gossypol plants with low- gossypol seeds. In this study, the new synthetic allotetraploid A_1_A_1_G_2_G_2_ also showed only very few glands on the cotyledon surface, which supported the analysis by Zhu et al [54]. Therefore, to develop varieties with the trait of high-gossypol plants with low- gossypol seeds, the dominant glanded gene, *Gl*_*3*_, in D subgenome of Upland cotton, should be replaced by *Gl*_*2*_^*b*^ from G genome. The obtained new synthetic allotetraploid between G. *herbaceum* and *G*. *australe* will allow us to have more choices of interested germplasm for development of high-gossypol cotton plants with low-gossypol seeds, which intrigues cotton breeders.

**Fig 8:**
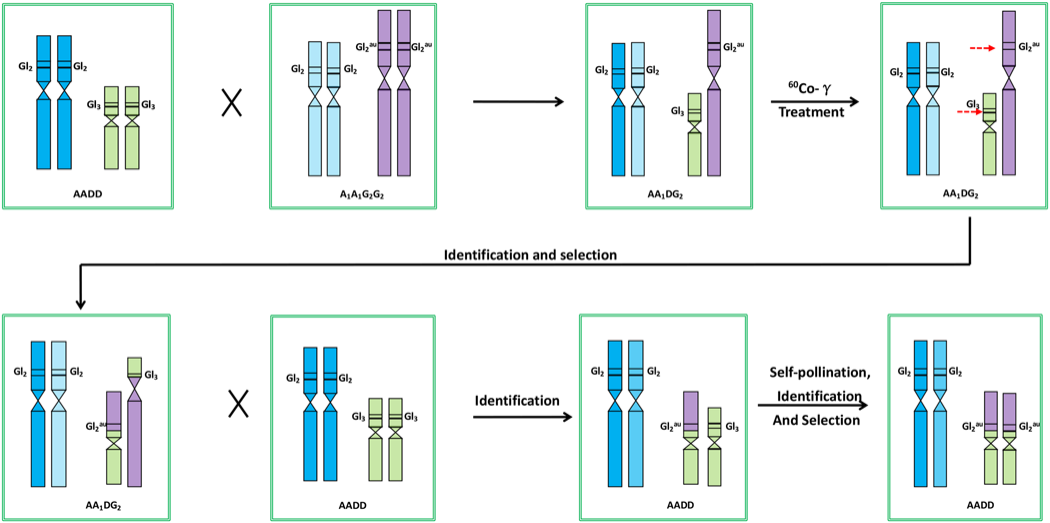
A strategy for the development of varieties with the trait of high-gossypol plants with low- gossypol seeds.

Here, we propose a new strategy for the development of varieties with the trait of high-gossypol plants with low- gossypol seeds to largely eliminate the role of *Gl*_*3*_ in conferring gossypol synthesis (Fig 8). Firstly, the A_1_A_1_G_2_G_2_ tetraploid is employed to cross with Upland cotton (AADD) to produce the tri-species hybrid (AA_1_DG_2_). For the obtained hybrid of AA_1_DG_2_, genetic recombination between A and A_1_ genome chromosomes often occur due to their close relationship while very little or no recombination occurs between D and G_2_ genome chromosomes under natural conditions due to distant relationship. To facilitate exchanges of D and G_2_ genome chromosomes, secondly, radiation should be employed on seeds or pollens of the tri-species hybrid (AA_1_DG_2_) to induce chromosome translocations between D and G_2_ genome chromosomes. Finally, the progenies derived from radiation inducement will be self-pollinated and characterized by combination of molecular cytogenetics, molecular markers and morphology to identify chromosome translocations between D and G_2_ genome chromosomes, particulary Chr. D12 translocated by 12G. If Chr. D12 is translocated by 12G and *Gl*_*3*_ is replaced by *Gl*_*2*_^*au*^ from *G*. *australe* via chromosome translocation, the translocation line should possess the traits of high-gossypol plants with low- gossypol seeds introgressed from *G*.*australe*. This research is under way.

## Supporting Information

S1 Fig Boll set on a chromosome-doubled branch of the hybrid F1 plant, *G*. *herbaceum* × *G*. *australe*, with the treatment of 0.10% colchicine for 24 h. (TIF)

S2 Fig Seeds and fibers of *G*. *herbaceum* (1), *G*. *australe* (2) and the new synthetic allotetraploid S_1_ (3). Bar = 25 mm.
(TIF)

S3 Fig S_1_ seedling (derived from the new synthetic tetraploid self-pollinated) at the second true leaf stage. (TIF)

S4 Fig S_1_ seedling became flavescent at the fifth true leaf stage. (TIF)
