# Supplementary figures and images for: A New Synthetic Allotetraploid (A1A1G2G2) between Gossypium herbaceum and G. australe: Bridging for Simultaneously Transferring Favorable Genes from These Two Diploid Species into Upland Cotton
Source: PLoS One. 2015 Apr 16;10(4):e0123209. doi: 10.1371/journal.pone.0123209 (PMC4400159; doi:10.1371/journal.pone.0123209)

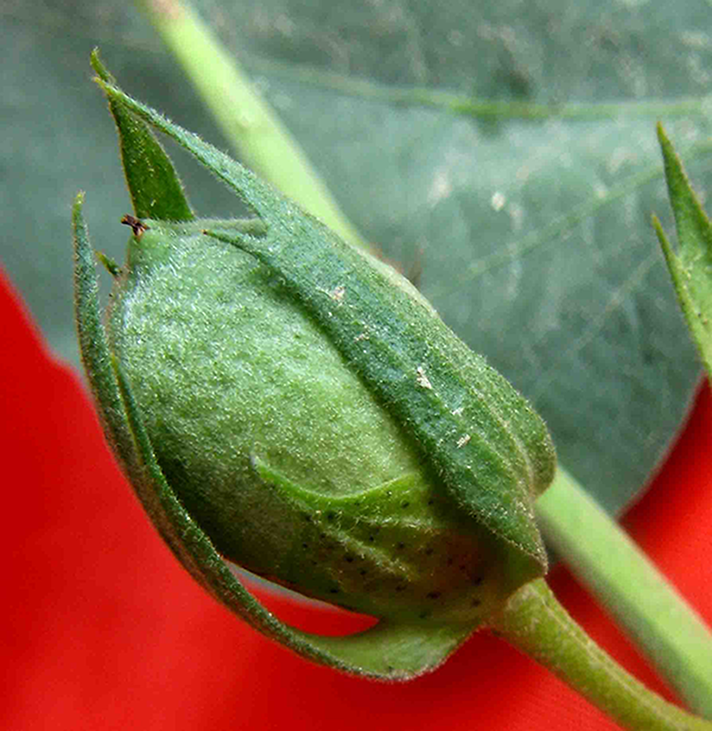

Supplement: S1 Fig — (TIF) [file pone.0123209.s001.tif]

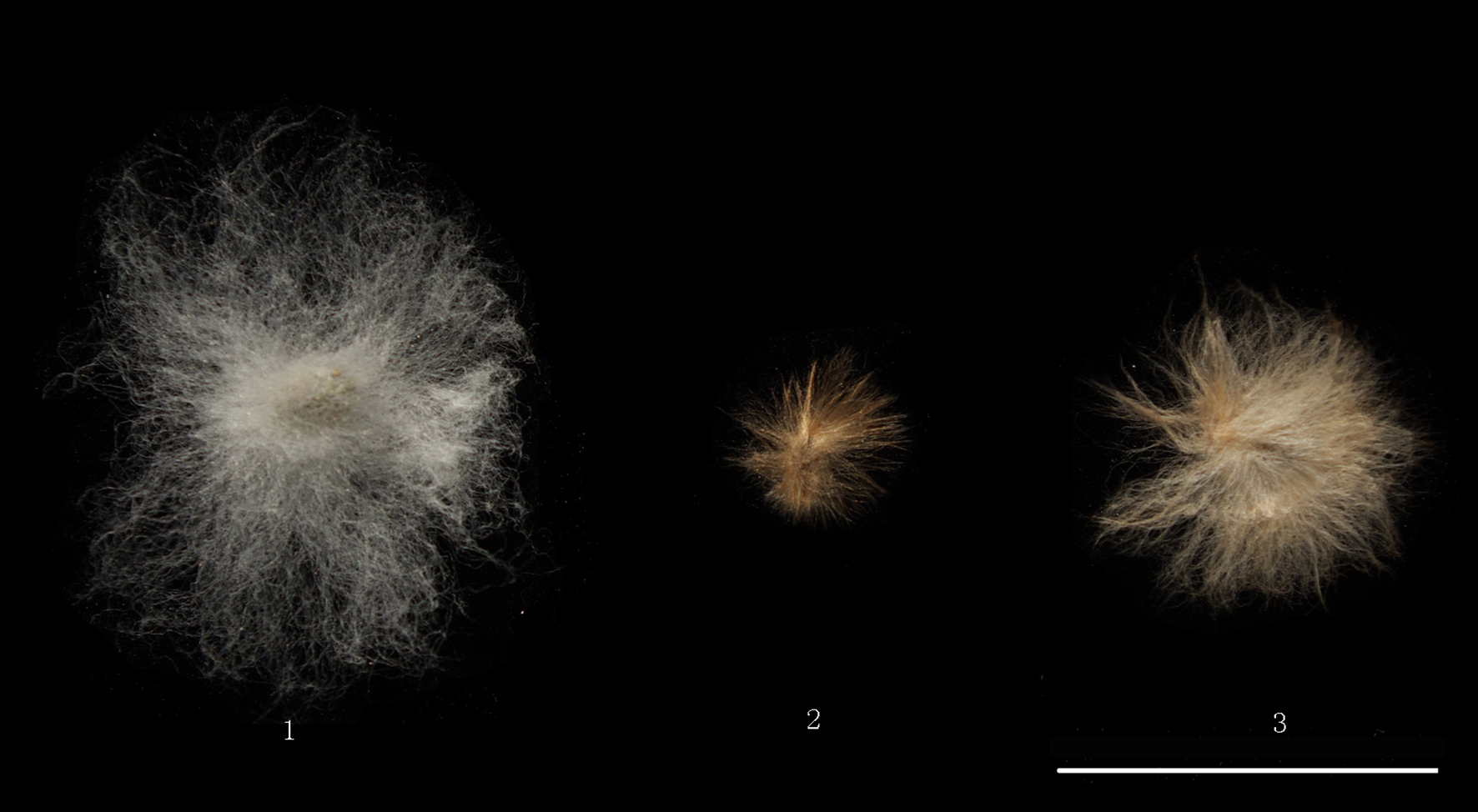

Supplement: S2 Fig — Bar = 25 mm. (TIF) [file pone.0123209.s002.tif]

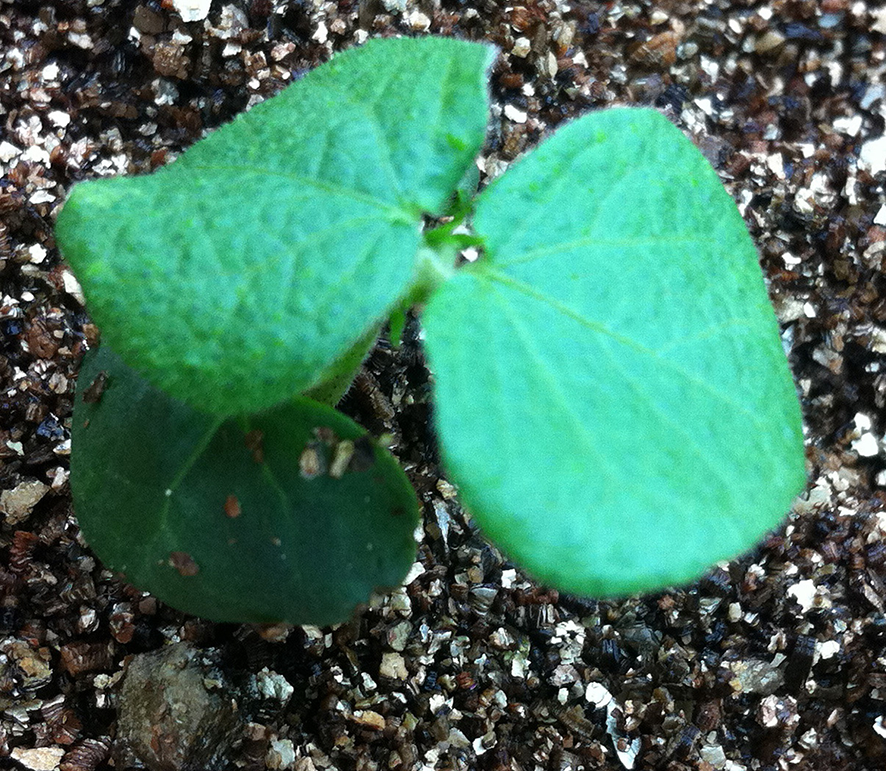

Supplement: S3 Fig — (TIF) [file pone.0123209.s003.tif]

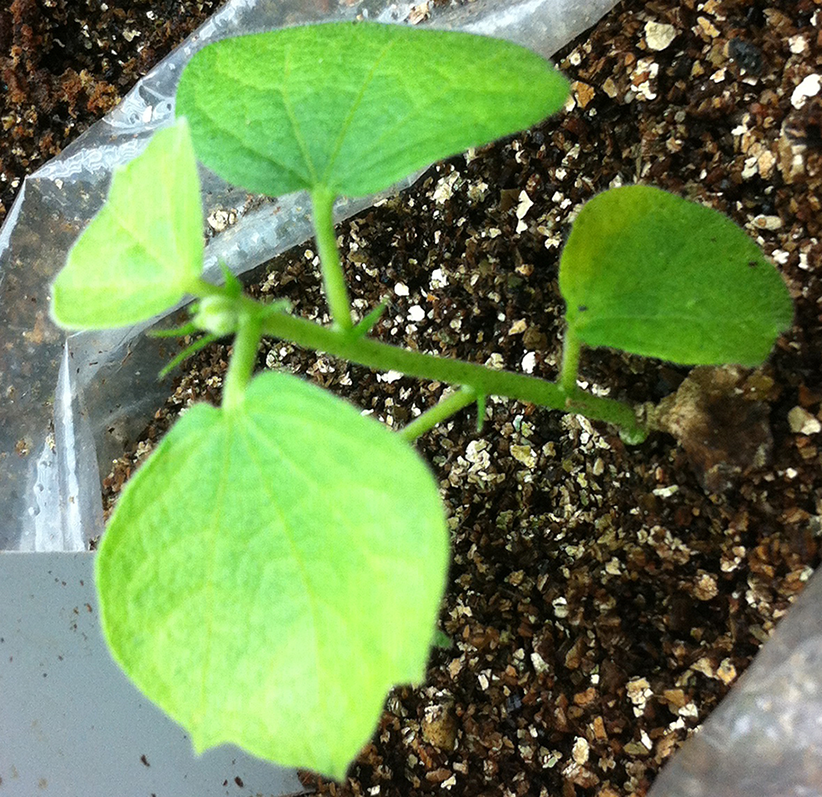

Supplement: S4 Fig — (TIF) [file pone.0123209.s004.tif]
